# A unified model for BAM function that takes into account type Vc secretion and species differences in BAM composition

**DOI:** 10.3934/microbiol.2018.3.455

**Published:** 2018-06-22

**Authors:** Jack C. Leo, Dirk Linke

**Affiliations:** Department of Biosciences, University of Oslo, Blindernveien 31, 0316 Oslo, Norway

**Keywords:** autotransport, protein secretion, Gram-negative bacteria, type V secretion, BAM, POTRA, TAM, outer membrane, transmembrane β-barrel, trimeric autotransporter adhesin

## Abstract

Transmembrane proteins in the outer membrane of Gram-negative bacteria are almost exclusively β-barrels. They are inserted into the outer membrane by a conserved and essential protein complex called the BAM (for β-barrel assembly machinery). In this commentary, we summarize current research into the mechanism of this protein complex and how it relates to type V secretion. Type V secretion systems are autotransporters that all contain a β-barrel transmembrane domain inserted by BAM. In type Vc systems, this domain is a homotrimer. We argue that none of the current models are sufficient to explain BAM function particularly regarding type Vc secretion. We also find that current models based on the well-studied model system *Escherichia coli* mostly ignore the pronounced differences in BAM composition between different bacterial species. We propose a more holistic view on how all OMPs, including autotransporters, are incorporated into the lipid bilayer.

## 1. Introduction

The term “autotransporter” was coined originally by Thomas Meyer and coworkers [1], based on earlier work from the same group showing that IgA protease from *Neisseria* is secreted to the bacterial cell surface through the outer membrane apparently without the help of any other factors. They suggested that this secretion mechanism involves a C-terminal “helper” domain forming a pore, and an N-terminal domain that represents a “proform” of the protease that is later released by autoproteolysis [2]. This work started a whole research field, and different groups were able to show that autotransporters can secrete even heterologous “passengers” to the cell surface, as long as they do not fold prematurely during their passage through the periplasm [3],[4].

While the “classical” autotransporters, with IgA protease from *Neisseria* being the prototype, were studied in much detail to understand the mechanism of secretion, it soon became apparent that there is a whole set of protein families with similar properties in Gram-negative bacteria, collectively termed Type V secretion systems [5],[6]. Please note that, while traditionally bacterial secretion systems are labelled using Roman numerals (example: “type Va secretion”), there is a recent tendency in the literature to use Arabic numerals in abbreviations (example: “T5SS” for type V secretion system). We (among others) prefer to use the traditional notation with Roman numerals. All type V secretion systems utilize the Sec machinery to pass the inner membrane with the help of a cleavable signal peptide, and then form a β-barrel pore in the outer membrane to export a passenger domain or protein. However, there are major differences between the different type V subclasses. In the current classification system, classical autotransporters represent the type Va secretion systems. They have a 12-stranded β-barrel translocator domain at their C-terminus that forms a transmembrane pore, and an N-terminal passenger that is exported through that pore (or so the prevailing model postulates). Type Vb secretion systems, or two-partner secretion systems [7], have their passenger and translocator encoded as two separate genes in an operon. The translocator of the type Vb systems is a 16-stranded β-barrel belonging to the Omp85 family and has additional periplasmic domains of the POTRA type (for “polypeptide transport-associated”—see below). Type Vc secretion systems are trimeric autotransporters, where three chains contribute four β-strands each to form the 12-stranded translocator pore; in this case, the passenger is a highly intertwined trimeric structure [8]. The type Vc secretion systems are also termed trimeric autotransporter adhesins (TAAs), as almost all examples described so far have been shown to have adhesive functions [9]. Type Vd secretion systems have a C-terminal translocator domain and an N-terminal passenger domain just like the type Va systems, but they are separated by a POTRA domain, making them look like a fusion of the two genes of a two-partner secretion system (or a “hybrid” of Type Va and Vb) [10]. All characterised passengers of type Vd systems are patatin-like phospholipases, but their secretion pathway has not been extensively studied [10],[11]. Last but not least, it was only recently recognized that there are autotransporters with an N-terminal translocator domain and C-terminal passenger, the so-called “inverse autotransporters” or type Ve secretion systems [12]. They differ from type Va systems in their domain order, but also in the type of passengers that they export: while type Va passengers are often β-helical structures, type Ve systems export repeats of small globular domains called bacterial Ig-like domains.

Current research strongly suggests that the term “autotransporters” for the type V secretion systems is misleading, and that these proteins actually rely on multiple external factors for their export to the bacterial cell surface, for different steps on the way. These factors include the aforementioned Sec machinery for inner membrane translocation, periplasmic chaperones such as SurA, Skp and others that have been shown to keep autotransporters in an export-competent state in the periplasm, and last but not least the β-barrel assembly machinery (“BAM”), one of the few essential outer membrane components in Gram-negative bacteria that catalyzes the membrane insertion of practically all outer membrane β-barrel proteins, which includes the autotransporters.

## 2. BamA models and function

In *Escherichia coli*, the BAM is a heteropentameric complex consisting of a central β-barrel subunit, BamA, and four associated lipoproteins, BamBCDE [13],[14]. Of these, only BamA and BamD are essential, but deletions of the other lipoproteins lead to defects in outer membrane homeostasis [13],[15],[16]. Like the β-barrels of type Vb secretion systems, BamA belongs to the Omp85 family and contains a 16-stranded β-barrel and five periplasmic POTRA domains in *E. coli*. Substrate outer membrane β-barrel proteins (OMPs) are recognised by a conserved motif residing in the C-terminal β-strand of the β-barrel domain [17].

**Figure 1.**
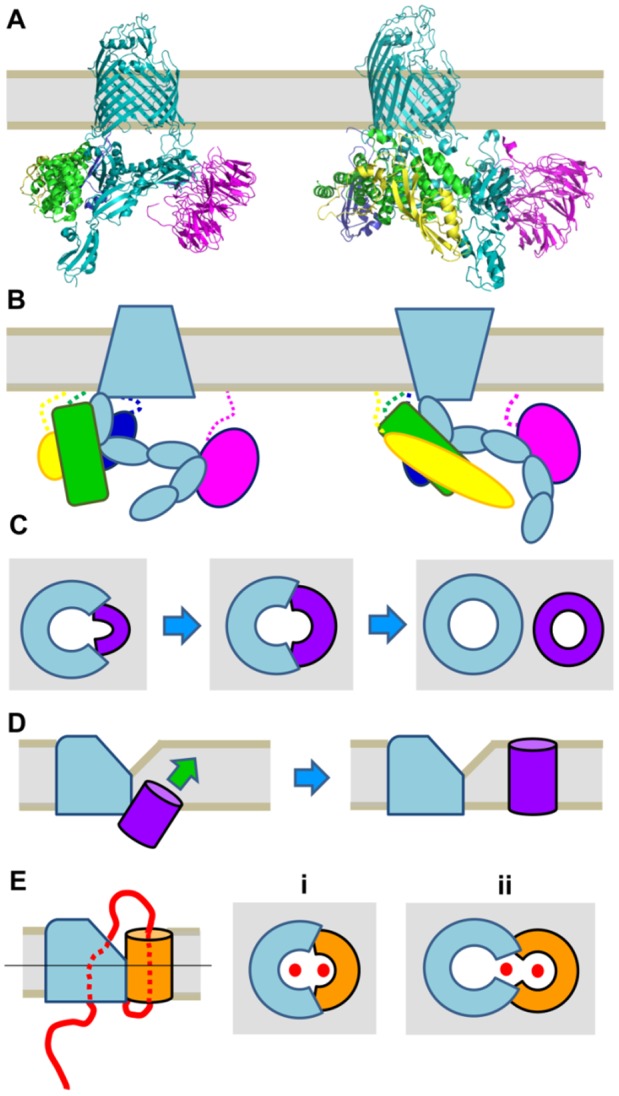
Function of the BAM complex. (A) Structures of the BAM from *E. coli*. The crystal structure on the left (PDB ID 5AYW) shows the “inward open” conformation, whereas the cryo-EM structure on the right (PDB ID 5LJO) shows the “outward open” conformation. BamA is in light blue, BamB in magenta, BamC in yellow (only the N-terminus is visible in 5AYW), BamD in green, and BamE in dark blue. The approximate position of the membrane is shown in grey. (B) Schematic representation of the structures shown in A. The colouring is the same. To emphasise the conformational changes in BamA, the wider end represents the “open” part, whereas the narrow end is “closed”. Dashed lines represent connection from the lipoproteins to the membrane. (C) Schematic representation of the budding model of BAM function. The view is a cross-section in the membrane plane (grey). The substrate OMP (violet) is inserted hairpin-wise into the lateral gate of BamA (light blue). Once the entire OMP is inserted, the β-barrel closes and detaches from BamA releasing the mature OMP into the outer membrane. Based on [37]. (D) Schematic representation of the assisted model of BAM function. The view is from the side. POTRA domains and lipoproteins are not shown for clarity. The substrate OMP (violet) inserts at the lateral gate, where membrane pinching causes local disorder of the lipids and facilitates the insertion. The green arrow shows the direction of movement; blue arrows denote the sequence of events. Based on [37]. (E) Schematic representation of how BamA might assist in autotransport. The side view (on the right) shows BamA (light blue) interacting with a nascent autotransporter. The β-barrel of the autotransporter is in orange, and the (unfolded) passenger is in red; the dashed parts of the passenger are inside the lumen of the β-barrel(s). The line represents the cross-section shown in the next two images, which represent alternative models for BamA-mediated autotransport: (i) in the hybrid model, BamA and the autotransporter form a hybrid β-barrel, the lumen of which serves as the secretion pore for the passenger, (ii) whereas in the tailored open barrel model, the autotransporter β-barrel stays in a partly open conformation that is prevented from closing by the passenger and the linker. BamA stabilises the partly open barrel and accommodates bulky parts of the passenger. The models are based on [35].

Over the past few years, several structures of BamA, either alone or in complex with assorted lipoproteins, have been solved [18]–[23] (Figure 1A). All the structures of the complex are from *E. coli*. BamA has two notable features: firstly, the seam of the β-barrel between β-strands 1 and 16 is destabilised, and the two strands can dissociate to create a lateral opening [18]–[20]. This opening may be modulated by the accessory lipoproteins [19],[22]. Secondly, the rim of the β-barrel is significantly narrower at this site than the opposite side of the β-barrel, leading to a local perturbation of the outer membrane at this site [18],[20]. A third feature of BamA is the so-called “exit pore” on the extracellular side of the β-barrel above the seam of the barrel [24]. In the various crystal structures, the exit pore is either in a closed or an open state, depending on whether the lateral gate is also closed or open, respectively. This pore has been proposed to accommodate extracellular loops or domains of substrate proteins during OMP biogenesis [24].

The picture of BAM function is further complicated by the POTRA domains and the accessory lipoproteins. The interactions with the lipoproteins are mediated by the POTRA domains of BamA. In the structures of the BAM, the POTRA domains are arranged as a ring around the BamA β-barrel, with the lipoproteins arranged on the outside of the ring, making the periplasmic part of the complex a funnel-like structure leading to the β-barrel of BamA [19],[20]. Structural and biochemical analyses have shown that the POTRA domains are dynamic and can alternate between different conformations [25]–[27]. Notably, POTRA 5, proximal to the β-barrel domain of BamA, can either occlude the lumen of the barrel on the periplasmic side, or swing out to provide access [19]. Despite a wealth of structural and biochemical data, how the BAM functions to insert OMPs into the outer membrane is still poorly understood. The available structures have given rise to a number of models for BAM function. Currently, two models are considered viable. In the first, called the “budding” model, nascent OMPs are inserted into the membrane environment through the lateral gate of BamA, one β-hairpin at a time (Figure 1C). This gives rise to a hybrid β-barrel, where BamA accommodates the β-hairpins by hydrogen bonding its β-strands at the seam (strands 1 and 16) to the β-strands at the edges of the OMP. Once all the β-strands of the OMP have been inserted, the β-barrel domain, which has been held open by binding to BamA, closes and buds off BamA to enter the outer membrane. In the second model, termed the “the BAM assisted model”, substrate proteins insert more or less spontaneously into the outer membrane (Figure 1D). In this model, the role of the BAM is to provide a conduit for the OMPs to reach the outer membrane, and the thinning of the membrane at the seam between β-strands 1 and 16 of BamA destabilises the membrane, thus providing an entry point for the nascent β-barrel protein. Both models are supported by biochemical data [24],[28],[29]. Of particular note is a recent study examining the function of Sam50, the mitochondrial homologue of BamA [30]. In this paper, a series of elegant crosslinking experiments provided clear evidence for the formation of a hybrid β-barrel, and also suggested that both the budding and assisted models may be at play simultaneously.

## 3. Autotransporters and BAM

To what extent does the BAM contribute to autotransporter function? Autotransporter biogenesis definitely depends on BamA, as shown for type Va [31],[32], Vc [33] and Ve secretion systems [34]. In all of these cases, evidence is indirect, either from experiments where the essential protein BamA is depleted from cells, or the BAM recognition motif is modified in the autotransporter, or both. These modifications always lead to less membrane insertion, reduced surface display, and often protein degradation through the periplasmic stress responses.

A multitude of crosslinking experiments have been performed, mostly on classical (type Va) autotransporters, (reviewed in detail in [35]). In summary, the results of these studies show that BamA and all other components of the BAM interact not only with the barrel part of the nascent autotransporter, in good accordance with general β-barrel protein insertion models, but importantly also with parts of the passenger domain (e.g. shown in [36]). This has led to two different models for autotransporter membrane insertion by the Bam complex: one where the (type Va) autotransporter C-terminal domain is fused to the BamA barrel, yielding a larger hybrid barrel with enough space to secrete the passenger (and in line with this model for β-barrel biogenesis: [37]), and a second one where the (type Va) autotransporter barrel is mostly formed but not completely closed, and is stabilized by BamA laterally while the transport of the passenger proceeds [36] (Figure 1E).

## 4. Species differences in BAM

**Figure 2.**
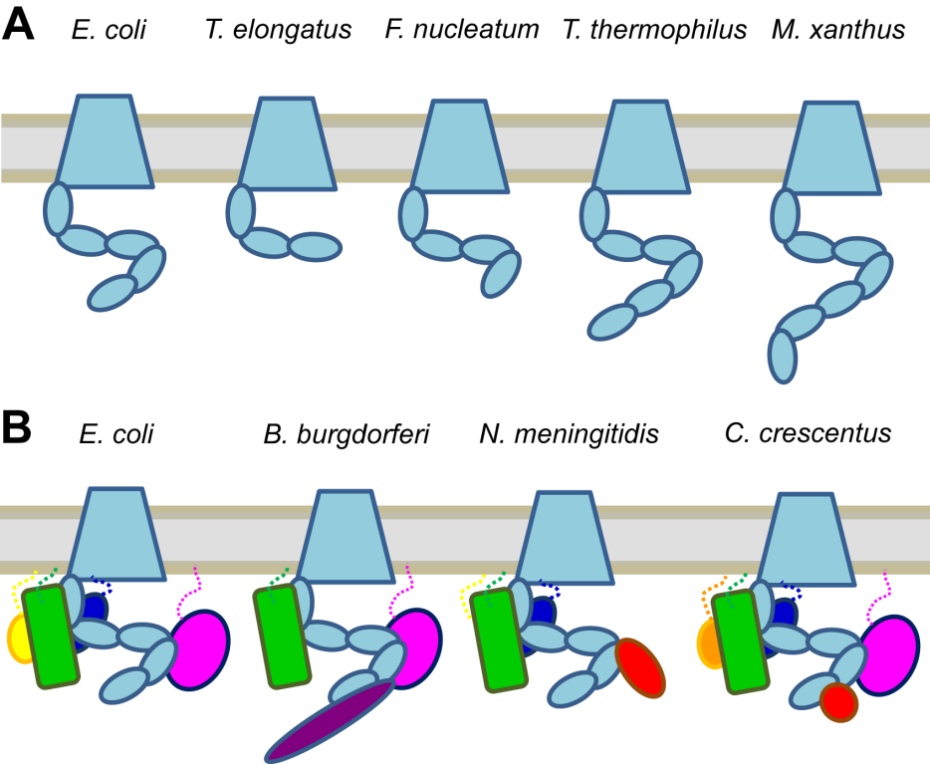
Diversity of BAM complexes in different bacterial species. (A) The number of POTRA domains in BamA differs between bacterial phyla, and some examples are depicted schematically here. In *E. coli* (*γ-Proteobacteria*) and related species, the number is 5. BamA from *Cyanobacteria* (exemplified by *Thermosynechocystis elongatus*) contain 3 POTRAs, whereas in *Fusobacterium nucleatum* (*Firmicutes*) it has 4, in *Thermus thermophilus* (*Deinococcus-Thermus*) it has 6 and *Myxococcus xanthus* (*δ-Proteobacteria*) has 7 [38]. (B) The accessory proteins of BAM can also differ between species, with some examples shown here schematically. *E. coli* has 4 accessory lipoproteins, BamABCD, whereas *Neisseria meningitidis* lacks BamB, but contains a non-lipoprotein constituent (RmpM). In *Caulobacter crescentus*, BamC is replaced by the non-homologous BamF. In addition, a soluble protein (Pal) is associated with the *C. crescentus* BAM. BAM of *Borrelia burgdorferi* lacks BamCE, but interacts with TamB [45],[46]. The colouring in the figure corresponds to Figure 1: BamA is shown in light blue, BamB in magenta, BamC in yellow, BamD in green, and BamE in dark blue. BamF is shown in orange, TamB in purple, and accessory soluble proteins (RmpM, Pal) in red.

When discussing the BAM and its function, it is easy to forget that this complex is quite different in different species—adding an extra layer of complexity to the discussion. Structures of the N-terminal periplasmic extensions of the BamA homologues in different cyanobacterial species show only three POTRA domains compared to the five in *E. coli* BamA [38],[39] and bioinformatics analysis shows that the number of POTRA domains can vary from 1 to 7 in different Gram-negative species [38] (Figure 2A). Likewise, the accessory lipoproteins of the Bam complex mostly do not exist in cyanobacteria, and only in part in some other species. In *Neisseria meningitidis*, for example, BamB is missing but a different lipoprotein [40] and an additional non-lipoprotein are involved in β-barrel insertion and assembly [41] (Figure 2B). When looking at the lipoprotein distribution in different species more systematically, only BamA itself and BamD are conserved [42], while the other lipoproteins differ from species to species, leading to the question whether *E. coli* is a good model for assessing Bam complex function in general, or whether species with simpler core complexes should be used to understand the basic functions of the BAM better. Differences in BAM composition, but also differences in the recognition motifs responsible for β-barrel protein insertion (see above) might explain why some outer membrane proteins are difficult to express heterologously in other species [43],[44].

## 5. TAM—an alternative insertion machinery for a subset of autotransporters

In addition to BAM, another complex is involved in the biogenesis of certain autotransporters, the Translocation and Assembly Machinery (TAM) [47]. TAM consists of two proteins: an Omp85-family outer membrane protein, TamA, and an inner-membrane anchored, periplasmic protein called TamB. TamA is homologous to BamA, and includes many structural features of BamA, including the narrow hydrophobic stretch and destabilised β-strand at the seam of the β-barrel, an exit pore and three periplasmic POTRA domains [48]. TamB is a large protein with a C-terminal region that forms a β-“taco” fold, which could potentially act as a chaperone or conduit for substrate proteins [49]. The C-terminus of TamB and the POTRA domains of TamA are the site of interaction, and this interaction is required for TAM function [50]. In a reconstituted system, the addition of a substrate autotransporter induced a movement of the POTRA domains and a change in the lipid environment around the TamA β-barrel, possibly mimicking the initial insertion steps of the autotransporter protein into the membrane [50].

TAM was discovered as a factor required for the biogenesis of a subset of type Va autotransporters in *E. coli*, including EhaA and Antigen 43 [47]. In addition, TAM also plays a role in the assembly of chaperone-usher pili [51]. However, the involvement of TAM in autotransport is not universal, as some other autotransporters including Hbp and EspP are efficiently secreted even in the absence of TAM [32],[52]. Furthermore, Antigen 43 can be secreted in an *in vitro* reconstituted system containing only BamA, demonstrating that TamA is not required in this setting [53]. TamA was shown to have a small effect on the folding of the type Ve autotransporter intimin, but its role in the biogenesis of type Ve secretion systems has not been studied more widely [54]. Recent data from our laboratory shows that TamA is not required for biogenesis to two type Vc-secreted proteins, YadA and EibD (Saragliadis et al., *J Vis Exp*, in press). The effect of TamA on Type Vb and type Vd systems has yet to be tested.

Though it is widespread within the proteobacterial phyla, TamA is not universally distributed [55]. However, unlike BamA, when present the number of POTRA domains in TamA does not vary between species. In addition, species within the *Bacteroidetes* and *Chlorobi* lack TamA itself, but contain a TamA homologue with a lipoprotein signal at the N-terminus, termed TamL [55],[56]. Interestingly, there are examples of organisms that lack TamA (and TamL) but do contain a *tamB* gene. In these cases, *tamB* is often found together in an operon with *bamA*, suggesting that in these organisms, the two proteins interact [56]. This interaction has been demonstrated in the case of *Borrelia burgdorferi* [46]. This diversity again underscores the complexity of the Omp85 family and its interactions with other proteins, including autotransporters.

## 6. Discussion

With only few exceptions that probably arose through convergent evolution, all OMPs in Gram-negative bacteria, including all autotransporters, are homologous [57], and this homology signal is strong enough to use for detecting and classifying OMPs (e.g. [58]). The evolutionary conservation extends to the C-terminal membrane insertion motif that is recognized by BAM [43]. To us, this conservation of the substrates calls for a universal model of the BAM mechanism, rather than for various models that only satisfy experimental data obtained from single subfamilies of outer membrane proteins (and/or autotransporters) individually.

OMPs, and especially the small OMPs with only 8 or 10 β-strands, can efficiently fold into artificial lipid bilayers and even detergent micelles in the absence of BAM *in vitro* [59]. All current models for BAM function suggest that it has a chaperone-like function. In analogy to enzymes, BAM catalyzes a process (in this case protein folding) that would in principle also occur in its absence—and as for any enzyme, we can assume it does so by lowering the activation energy of the process, thus speeding it up considerably. Small OMPs fold into lipid bilayers in a one-step, concerted process where all β-hairpins transverse the bilayer in a flipping motion to form the final structure [60]. BAM presumably assists in this process by stabilizing folding intermediates or by destabilizing the lipid bilayer to allow for easier insertion—or both.

A key problem of all current models for BAM function (as displayed in Figure 1) is that none of them fully explains how this would work for Type Vc secretion systems, the TAAs. TAAs, like all other transmembrane β-barrel proteins, depend on BAM for membrane insertion [33]. They form a barrel made from three subunits, each contributing four β-strands to the 12-stranded transmembrane β-barrel pore. All current data indicates that autotransport for TAAs proceeds via a three-hairpin intermediate through that pore ([61], Chauhan et al., under revision), similar to the hairpin intermediates shown for classical [62] and inverse autotransporters [34]. There is no experimental data that even remotely suggests sequential membrane insertion of the three units. In fact, recent data from the Bernstein lab suggests trimerisation of the β-barrel already in the periplasm [63]. Such “protobarrels” that are not fully inserted into the outer membrane have also been described for type Va autotransporters [32],[64].

The presence of periplasmic helper proteins that are themselves symmetric trimers, such as the inner membrane lipoprotein SadB that is needed for efficient surface display of the TAA SadA from *Salmonella* [65] or the peptidoglycan-binding protein TpgA that helps in surface display of the TAA AtaA from *Acinetobacter* ([66], and K. Hori, personal communication), rather suggest a mechanism where a pre-formed or at least pre-arranged trimer inserts into the outer membrane prior to autotransport. Similarly, it is hard to conceive how the highly intertwined structure of the TAA passenger domain could fold efficiently if the passenger chains are not exported simultaneously, but one after the other—and if the source of energy for autotransport indeed is exclusively the free energy of passenger folding, sequential passenger export cannot work as the highly intertwined TAA domains can only fold as trimers [8],[67]. Evidence for concerted folding of trimeric domains in the TAAs comes from detailed structural studies of the coiled-coil stalk that can transition from left- to right-handed supercoils, which would allow for relief of torsion stress during concerted folding and export [68],[69].

**Figure 3.**
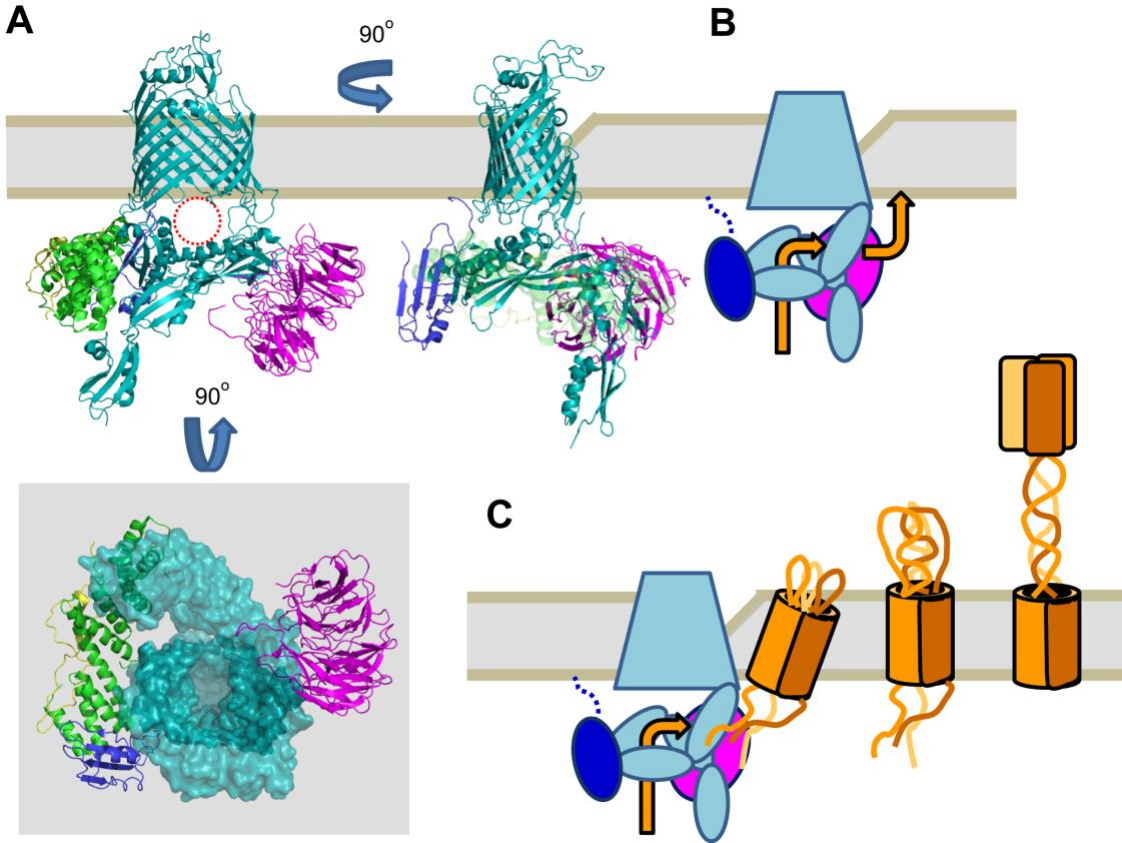
Model for insertion of TAAs into the outer membrane by BAM. (A) BAM has a funnel-like structure that can shuttle substrate OMPs to the site of insertion at the pinched membrane. Top right: the same view of BAM as in Figure 1A, right panel. The cavity below the seam of the barrel is highlighted with a dashed red circle. Top left: The structure from the right rotated 90° perpendicular to the membrane, demonstrating the membrane pinching and the cavity below. BamC and BamD have been made transparent for clarity. Bottom: The structure from the top right rotated 90° in the plane of the membrane, showing the funnel-like structure of BamA. BamA is shown as a space-filling model, and the POTRA domains have been made slightly transparent. (B) Schematic of the top left view in A. BamC and BamD have been omitted for clarity. The orange arrows demonstrate the pathway for the substrate to reach the outer membrane. (C) Model for insertion and folding of TAAs. A trimeric TAA “protobarrel”, including hairpins, is formed in the periplasm. This is transferred through the funnel of BAM to the OM, where the β-barrel is inserted and adopts its final conformation. Concomitantly, secretion of the passenger is initiated. Folding of the obligate trimeric passenger pulls the unfolded regions of the passenger through the β-barrel pore until the entire passenger is folded and exported.

While all the experimental evidence that gave rise to the different BAM function models in Figure 1 is sound, all of these current models do not satisfy the need of TAAs to insert into the lipid bilayer as partially preformed trimers in a concerted fashion. We propose here to merge the current models, and doing so, to accept that the individual steps of the mechanism of BAM captured by either high-resolution structural biology or low-resolution methods such as chemical crosslinking are only glimpses of a much more fluid, concerted process. In such a process, BAM would act as a periplasmic funnel to accept substrates that are recognized through their C-terminal insertion signal (Figure 3A). The substrates would pre-form into a membrane insertion-competent state while in the funnel, and would be pushed through a lateral opening into an area of the lipid bilayer that is significantly destabilized by the lateral gate of BamA (Figure 3B). The requirement for trimerisation before β-barrel insertion could explain why the attempted (and presumably abortive) heterotrimerisation of two different TAAs is lethal to the cell: poorly trimerised substrates cannot be inserted into the membrane, but are protected from DegP-mediated degradation in the funnel, thus effectively blocking the pathway for the insertion of other OMPs [70].

The motion of opening and closing of BamA, and the motions of the BAM lipoproteins in the periplasm, would all contribute to transient chaperoning of the substrate and to membrane insertion, without forming any substantial stable structural intermediates. The latter point is essential: large-scale formation and breaking of hydrogen bonds would probably not speed up, but rather slow down the process of insertion. The energy required for insertion is freed as the β-barrel membrane protein folds into its final structure. In the case of autotransporters, the tailing passenger domain could then squeeze through the transmembrane pore of the TAA itself. This model is shown schematically in Figure 3C. Note that for this model, it is of little consequence whether the autotransport hairpin is formed before, during or after membrane insertion of the barrel. We would rather see this as one fluid motion through the periplasmic funnel into the membrane, with distinct ratcheting only later as individual parts of the passenger start to fold on the cell surface, providing the necessary energy to pull out the rest.

## 7. Conclusions

Such a unified model would work for Type Vc TAAs, and it does not contradict any of the experimental crosslinking evidence (mainly for Type Va autotransporters), where both the barrel domain and the passenger are shown to interact with various parts of BAM, including most of the inner surface of the “funnel” and BamA itself. Such a mechanism also does not dispute the importance of the motions of a lateral opening or closing of the BamA barrel, which to our understanding helps in keeping the membrane import-competent locally. What it does contest is the claims of substantial hydrogen bond formation (and breaking) that would be necessary to form hybrid barrels (Figure 1C)—this cannot work for trimeric autotransporter adhesins, as it would require sequential insertion of all three units. An alternative model, where three BAM complexes act in unison to insert a single TAA β-barrel seems equally unlikely to us. Last but not least, this unified model can easily be adapted for different species, where then the exact nature of the periplasmic funnel would vary with the presence or absence of different BAM lipoproteins, and with different numbers of POTRA domains on BamA, while the general principle of recognition and chaperoning would remain the same. When discussing BAM function, it is crucial not to over-emphasize the importance of poorly conserved parts of the machinery.
